# PReS-FINAL-2049: Bone health, muscle strength, activity

**DOI:** 10.1186/1546-0096-11-S2-P62

**Published:** 2013-12-05

**Authors:** E Sandstedt

**Affiliations:** 1 Departement of Physical Therapy, Sahlgrenska Acedemy/ Quenn Silva children's Hospital, Gothenburg, Sweden

## Introduction

Juvenile idiopathic arthritis (JIA) effects bone health, muscle strength, physical fitness and well-being in children and adolescent. Due to biological medical treatment there is increased outcome of the disease with less flares and arthritis. Impaired bone health, muscle strength and decreased well-being have been reported in this group. There is evidence for intervention studies with cardiovascular fitness, physical fitness and muscle strength exercises for increased outcome of the disease.The aim was to evaluate an exercise programme in children and adolescents before and after 12 weeks with rope-skipping and muscle strength exercises and to evaluate the effect on bone health, muscle strength, physical fitness and well-being.

## Objectives

54 subjects participated, age 9-21 years, randomized into an exercise and a control group.

## Methods

A randomized controlled study.

DXA, grip-it, myometry, physical fitness and the questinnares CHAQ and CHQ were used. The exercise group performed the exercise programme in 12 weeks and physical activity in leisure time was documented in two 12-week diaries for both groups.

## Results

Bone health was within the normal in this group at base line and there were no differences between the exercise and the control group. Bone health, calculated as BMD increased in the exercise group after 12 weeks. Muscles strength showed values within the normal. Muscle weakness was found in hip extensors and hip abductors at base line and increased after the training period. Muscle weakness in the hand was shown in > 50% of the participants and there was no change during the study. Muscle strength in quadriceps was within the normal, increase after 12 weeks exercise and was maintained at follow-up. Pain and well-being were reported in the group.

## Conclusion

Bone health and muscle strength were within normal range at base line except in hip extensors, hip abductors and in hand grip strength. Muscle strength in knee extensors increased and was maintained at follow-up. Muscle strength training with free weights and rope skipping (100 jumps 3 times/ week in twelve weeks) increase BMD and muscle strength and can be recommended for children and adolescents with JIA. The presence of pain was reported from many children and needs to be addressed in view of pain in itself, and in view of the consequences of pain on well-being in psychosocial terms.

## Disclosure of interest

None declared.

